# Advancement in Diagnosis, Treatment, and Vaccines against *Fasciola hepatica*: A Comprehensive Review

**DOI:** 10.3390/pathogens13080669

**Published:** 2024-08-07

**Authors:** Pablo José Rufino-Moya, Rafael Zafra Leva, Álvaro Martínez-Moreno, Leandro Buffoni, Elora Valderas García, José Pérez Arévalo, Verónica Molina-Hernández, María T. Ruiz-Campillo, Guillem Herrera-Torres, Francisco J. Martínez-Moreno

**Affiliations:** 1 Animal Health Department (Parasitology and Parasitic Diseases), Faculty of Veterinary Medicine, University of Córdoba, Sanidad Animal Building, Rabanales Campus, 14014 Córdoba, Spain; pablo.rufino.moya@gmail.com (P.J.R.-M.); amm@uco.es (Á.M.-M.); h12bupel@uco.es (L.B.P.); sc2vagae@uco.es (E.V.G.); fjmartinez@uco.es (F.J.M.-M.); 2 UIC Zoonosis y Enfermedades Emergentes (ENZOEM), Faculty of Veterinary Medicine, University of Córdoba, Sanidad Animal Building, Rabanales Campus, 14014 Córdoba, Spain; an1pearj@uco.es (J.P.A.); vmolina@uco.es (V.M.-H.); v42rucam@uco.es (M.T.R.-C.); v82hetog@uco.es (G.H.-T.); 3 Departamento de Sanidad Animal, Facultad de Veterinaria, Universidad de León, 24004 León, Spain; 4 Department of Anatomy, Comparative Pathology and Toxicology, Faculty of Veterinary Medicine, University of Córdoba, Sanidad Animal Building, Rabanales Campus, 14014 Córdoba, Spain

**Keywords:** fasciolosis, ruminants, diagnosis, treatment, vaccines

## Abstract

In this review article, we aim to provide an overview of fasciolosis in ruminants. Diagnosis through new coprological methods (such as Flukefinder^®^, FLOTAC^®^, and Mini-FLOTAC^®^) remains the most suitable approach for farms. Regarding treatment, there is a scarcity of available drugs, and resistance to them has prompted new approaches (including drug combinations, enhanced metabolism, or the use of natural compounds) to address this issue. Additionally, several researchers have developed vaccines to control the disease, but their efficacy varies, and none are currently sufficient for commercial use. Further studies are needed to better understand all aspects discussed in this manuscript, with the goal of improving diagnosis, treatment, and disease control. It is important to note that this manuscript does not delve into in-depth knowledge of the discussed aspects; rather, it provides an overview of the different methodologies related to these three aspects of parasitic disease.

## 1. Introduction

Fasciolosis, caused by the trematode parasites *Fasciola hepatica* and *Fasciola gigantica*, has a worldwide distribution and is present in more than 81 countries [1,2]. This liver fluke affects a wide range of hosts, including domestic animals (such as cattle, sheep, goats, pigs, and horses) and wild herbivorous mammals (such as deer, rabbits, and hares), as well as humans [3,4]. The infection occurs through the oral ingestion of metacercariae, either by consuming uncooked or unwashed aquatic plants or by drinking contaminated water [5]. Actually, fasciolosis is considered an emerging zoonotic disease; according to the World Health Organization (WHO), 180 million people are at risk of infection, and between 35 and 72 million people are infected [2,6,7]. In ruminants, 350 million cattle and 250 million sheep are at risk of acquiring *F. hepatica*, posing a major problem in the farming industry due to substantial economic losses estimated at USD 3.2 billion per year [2,8,9,10]. These costs are the result of both losses in production (i.e., mortality, reduction in milk and meat production, fertility declines, or low quality of hides) and treatment with anthelmintic drugs [6,8,11].

Commonly, the control measures for *F. hepatica* infection primarily rely on the use of anthelmintic drugs. In this sense, the most common drug used has been triclabendazole due to its activity against both juvenile and adult forms of the parasite [3,12]. Due to this, triclabendazole has been extensively used in the livestock industry to prevent production losses. However, this practice has led to widespread resistance in cattle and sheep [13], appearing in triclabendazole-resistance strains of *F. hepatica* [7].

Bearing in mind this, and jointly with the changing climate that is responsible for the increasing prevalence of infection [4], there is an urgent need for alternative control methods as well as new diagnostic techniques able to detect early stages of infection.

Regarding the control methods, vaccines have been recognized as a promising and economically viable alternative strategy for controlling fasciolosis in livestock [14]. Numerous studies have been undertaken to boost humoral or cell-mediated immunity to fasciolosis through immunization with attenuated, recombinant, gene knockdown/silencing, nucleic acid-based vaccines, or a combination thereof [6]. These studies have utilized rats, mice, rabbits, and ruminants (cattle, sheep, and goats) as hosts [14]. Another potential alternative is the treatment with secondary compounds from plants with anthelmintic activity. In this regard, in vitro and in situ trials have demonstrated the flukicidal effect in ruminants of compounds derived from plants such as *Artemisia* spp., *Bassia latifolia*, *Moringa oleifera*, and other tropical species [8,15,16,17].

On the other hand, traditionally, the diagnosis of fascioliosis has been based on direct observation of *F. hepatica* at necropsy and abattoir findings [18] or through the observation of its dissemination elements. In this sense, coprological methods have been performed and implemented, but these methods are only useful in the late stages of the diseases [19,20].

Other techniques are immunologically based, have higher sensitivity, reproducibility, and cost-effectiveness, and have been useful since the early stages [21,22]. Lastly, in the last few years, with the “omics” era, molecular diagnostic methods have been developed to increase the sensitivity and specificity of conventional diagnostics at the same time that the detection time has been shortened [23,24]. Among other advantages, additionally, using these techniques, it is possible to detect mixed infections of *Fasciola* [25].

With all this background, the aim of this review is to offer updated information about the latest tendencies in diagnosis, treatment, and control through an effective vaccine against *F. hepatica*. To conduct this review, we retrieved references from PubMed using the advanced search mode. We employed Boolean operators (specifically “AND/OR”) and searched for the terms “fasciola”, “diagnostic”, “treatment”, and “vaccine”. Our search was restricted to the last 10 years to provide up-to-date information. Nevertheless, especially in the vaccines section, it was necessary to refer to older publications to establish an appropriate background.

## 2. Diagnosis

The detection of *Fasciola* is an essential component in disease control, given that the most significant pathogenic action occurs during the first weeks of infection when direct detection is not possible. This has driven the development of indirect techniques to identify *Fasciola* infection. Therefore, detection can be divided into two general modalities: direct and indirect.

### 2.1. Direct Methods

Direct diagnosis relies either on the microscopic identification of eggs or on the recovery of parasites during necropsy. While liver necropsy provides an accurate diagnosis of infection, it cannot be utilized as an antemortem tool for detecting active processes.

The typical direct antemortem test involves detecting parasite eggs in the host’s feces (Fecal Eggs Count—FEC), which is considered the ‘gold standard’ test [26]. This is a straightforward, non-invasive technique that yields immediate results. However, the coprological detection of the parasite is only feasible during the chronic phase (8–10 weeks post-infection), depending on the host species. Moreover, the test’s sensitivity can be influenced by various factors, such as the host’s age, fecal characteristics, egg-shedding rate, and parasite burden [27,28].

In the case of FEC, the test’s effectiveness is typically evaluated in terms of sensitivity and specificity. Sensitivity denotes the test’s ability to accurately identify infected individuals, while specificity refers to the test’s ability to correctly classify non-infected individuals as negative. Additionally, other parameters, such as ‘detection sensitivity,’ which refers to the lowest fecal egg load that can be reliably measured using a specific method, can be utilized [28].

The standard coprological method is sedimentation, which is routinely used in parasitology laboratories. The primary limitation of this technique, in addition to the pre-patency period, is its reduced effectiveness, particularly when parasite loads are low [29]. It can even result in false negatives, as reported in field studies [18,30,31].

Furthermore, *Fasciola* egg shedding has been observed to be intermittent, leading to significant variations in egg counts (EPG) over time [27]. When analyzing the ability to detect *F. hepatica* eggs in feces by routine sedimentation, it was observed that the sensitivity is 26% with values of 5 eggs per gram (epg), while the sensitivity increases to 100% when the sample contains 20 epg [32].

To overcome these limitations, various flotation–sedimentation-based techniques have been developed. These include the FLOTAC^®^, the Mini-FLOTAC^®^, and, notably, the Flukefinder^®^. The latter is a commercial egg detection device that employs a modified sedimentation and fine filtration technique.

Flukefinder^®^ appears to be the most accurate method for *Fasciola* egg detection, with a sensitivity of 80% and a specificity of 90% compared to sedimentation [33] and 100% in artificially spiked stool samples. When compared with Kato–Katz and Mini-FLOTAC^®^ in humans, it was observed that Flukefinder^®^ detected a lower egg burden and less variability compared to the other techniques [34].

In a recent study comparing routine sedimentation with Flukefinder^®^ and a modified version of Flukefinder^®^ involving double sedimentation, it was found that the sensitivity of the three methods was comparable. However, both Flukefinder^®^ and the modified Flukefinder^®^ were superior to sedimentation in terms of egg count data [35].

It is crucial to use a method capable of detecting low egg elimination rates. In this sense, a recent study comparing Mini-FLOTAC^®^, sedimentation, and Flukefinder^®^ found that the sensitivity of all techniques is 90% when the burden is 20 epg [36]. However, the modified Flukefinder^®^ achieves a maximum sensitivity of 5 epg, while Flukefinder^®^ can detect up to 2 epg. In the same way, Mini-FLOTAC^®^, using zinc sulfate as a flotation solution, yielded results inferior to those obtained with classical sedimentation [35].

In any case, it appears that for an egg burden greater than 20 epg, the various methods described below demonstrate a higher sensitivity than traditional coprological analysis in detecting *F. hepatica* eggs. From a clinical point of view, these types of methods are the best to perform on farms because they are easy to carry out, do not require specific instruments, and are highly useful for monitoring the status of farms and detecting the presence of infections.

A summary of these methods is shown in Table 1.

### 2.2. Indirect Methods

#### 2.2.1. Immunodiagnosis (ELISA)

An alternative to direct methods is immunological diagnosis. This method has been developed and adapted for diagnosis, proving to be highly reliable. It enables early detection of the infection during the pre-patent phase and allows for the analysis of many animals simultaneously, with results obtained rapidly. The drawbacks of this technique include the potential for cross-reactions with other parasites, as well as the cost of both the equipment and the reagents required to implement the technique.

The most prevalent technique today is the Enzyme-Linked Immunosorbent Assay (ELISA), which has superseded other techniques, such as immunoelectrophoresis, complement fixation, double diffusion, and haemagglutination, due to its relative complexity [37]. Considering ELISA as the benchmark technique for immunodiagnosis, several modifications of ELISA have been introduced, with two major variants. The first is an indirect ELISA, which is utilized for the detection of circulating antibodies against a specific antigen immobilized on the ELISA plate. The other is a sandwich ELISA, in which antigens against a known antibody fixed for diagnosis are detected.

##### Indirect ELISA

Indirect ELISA employs a two-step process. Initially, the primary antibody binds to the antigen, followed by incubation with a labeled secondary antibody. In relation to this, antibodies against *Fasciola* spp. can be detected in serum from 2 weeks post-infection [38], and they remain at detectable levels in various hosts for up to 20 weeks post-infection, even following the eradication of the parasites [37]. A crucial aspect in the development of an ELISA is the selection of the specific antigen. A wide range of products have been utilized, with secreted, natural, and particularly recombinant excretion products currently in use.

Crude excretory antigens, derived from parasite culture and subsequent filtration/purification of the culture fluid, have been trialed in cattle. Antibody concentrations (AC) were observed at 2–4 weeks post-infection (p.i.), and these levels were sustained for a minimum of 7 months [21]. In this way, we formulated crude antigen, excretory/secretory (E/S) products, and glutathione S-transferase (GST) from adult *F. gigantica* and evaluated their efficacy in cattle, sheep, and donkeys [39], with the most favorable sensitivity and specificity observed for the E/S antigen. It was noted that the production of the GST antigen entailed a cost and effort that was not justified by the results.

On the other hand, cathepsins are generally considered to be one of the antigens that yield the best results, as they likely stimulate the highest proportion of specific antibodies during experimental *Fasciola* infection [40]. This cysteine protease is secreted by the parasite throughout all stages of its development, participating in traversing the gut (cathepsin B, L3, and L4) [41], liver migration (cathepsins L1 and L2) [42], and finally into the bile ducts (cathepsin L1) [43]. It has been demonstrated to be highly immunogenic [26].

Within this group of cathepsins, which is composed of numerous molecules with minor variations in both composition and activity, several have been tested in immunological diagnostic tests in both their native and recombinant forms.

Among the types of cathepsins, recombinant precathepsins rFhpCL1, rFhpCL2, and rFhpCL5 have been tested against serum from experimentally infected sheep and cattle, achieving a sensitivity of 100% in sheep and 97% in rFhpCL2 in cattle [40].

Recombinant cathepsins (FhCL1, FhCL2, FhCL3, and FhCB3) have been tested, detecting positive antibody levels in experimentally infected sheep at 3 weeks post-infection, with higher values for FhCL1 and FhCL3. These levels were sustained throughout the 16-week duration of the experiment [44].

In assays with isolates, the cathepsin B5 ELISA demonstrated a sensitivity of 95.3% and a specificity of 92.4% in tropical bovine fasciolosis [45]. In the pursuit of new antigens, recombinant cathepsin CL7 has been tested, showing a sensitivity and specificity of 100% in sheep and 100% and 93.75% in cattle, respectively, and being able to detect infection in buffalo at 3 weeks post-infection [46].

Despite the high sensitivity and specificity of cathepsin tests, their development and application necessitate complex techniques that can complicate their production. Consequently, new antigens have been explored to facilitate the development of these immunological techniques. Kunitz-type (KT) protease inhibitors, which are low-molecular-weight proteins with serine protease inhibitory activity, play a role in the regulation of cathepsins. Within this group, FhKT1-type proteins have been adapted for use in ELISA, yielding positive results for immunodiagnosis [47].

In addition to cathepsins, other antigens have been utilized to detect the production of specific antibodies. Fatty acid binding proteins (FABPs) and glutathione S-transferase (GST) are considered immunogenic proteins that can be used for diagnostic purposes [48,49,50]. However, in a joint study with cathepsin L1, it was deemed the most suitable due to its sensitivity and specificity values [51].

Other products employed in indirect ELISA diagnostics include tegument antigens. These were adapted to commercial kits, but limitations arose due to cross-reactivity [37]. Similarly, antigens extracted from the purification of excretory/secretory (E/S) products have been tested in immunodiagnosis, achieving a sensitivity and specificity of 65% and 100%, respectively [52]. However, subsequent tests detected cross-reactivity with *Dictyocaulus* spp., resulting in a decrease in sensitivity and specificity to 65% [53].

##### Sandwich ELISA

This type of ELISA offers the advantage of detecting active *Fasciola* infections. It relies on identifying circulating antigens that bind to monoclonal antibodies immobilized on the plate. These ELISA tests can be used with serum, milk, or fecal shed antigens (coproantigens). Commercial kits have been developed based on this approach, allowing for the early detection of active infections with high sensitivity and specificity [48,54].

Among the antibodies used, MM3, a fraction purified from *Fasciola* E/S products, has been adapted in various variants of indirect ELISA. It has been tested in serum and milk, both in individual samples and bulk tank milk [55]. In serum samples, the sensitivity and specificity were 99.2% and 100%, respectively, while in individual milk samples, they were 93.9% and 98.1%. The correlation between serum and milk remains valid even in bulk milk samples [54]. The same antibody has also been adapted for detecting coproantigens [56], with modifications that significantly enhance its specificity and sensitivity [57]. In field tests, a strong correlation (and increasing sensitivity) with coprological techniques has been demonstrated, which disappears when the parasite is eliminated through treatment [58].

In addition to the widely used MM3 antibody, other tests based on monoclonal antibodies against *Fasciola* have undergone evaluation in various assays. One such test employs antibodies (AB) against *F. gigantica* cathepsin-like antigens to detect circulating cathepsin CL1 and CB3 in bovine assays. This test detects the antigen as early as 1 day post-infection (d.p.i.) and concludes that it can be utilized for the early diagnosis of parasitism [48,59].

On the other hand, considering the advantages of immunodiagnostics, such as early diagnosis and the ability to test many samples in a short time, several commercial kits based on both antigen and antibody detection have been developed. These kits include Ildana Biotech^®^, IDEXX^®^, Svanova^®^, and Bio-X^®^. A recent study examined these four products and concluded that Ildana^®^, IDEXX^®^, and Bio-X^®^ are valuable for detecting active *Fasciola* spp. infections. Bio-X^®^ is effective for treatment control, and all these kits serve as suitable tools for monitoring *F. hepatica* infection through the analysis of single bulk tank milk samples [60].

Summing up, immunological diagnosis represents a significant advancement in the early detection of infections. However, it still has limitations, including the possibility of false negatives or false positives, the persistence of antibodies in serum even after the parasite has cleared, and the restricted detection capability of coproantigens to active infections [29,56]. Generally, from a clinical point of view, these methods exhibit high sensitivity but are confined to diagnosing established infections within the host through direct sampling via blood and/or fecal collection. Additionally, the high cost of immunodiagnostics, estimated at approximately AUD 22 per sample, along with the need for specific equipment, compounds these limitations [61].

### 2.3. Molecular Techniques

The detection of *Fasciola* DNA using various molecular methods has been employed for diagnostic and speciation purposes. The primary advantage of these molecular techniques lies in their ability to identify the specific species involved in the infection, which is particularly relevant in areas where both *F. hepatica* and *F. gigantica* coexist. However, a significant limitation of these methods is that the source of DNA is the parasite eggs [58].

In this context, several techniques have been developed. For instance, results obtained through single-step duplex PCR enable rapid and accurate identification [25]. Additionally, the development of RT-PCR for species identification has demonstrated high sensitivity and a strong correlation with fecal egg counts. When comparing multiplex PCR and PCR-RFLP, multiplex PCR has consistently outperformed RFLP in terms of results [62].

In Iran, the use of multiplex PCR and PCR-RFLP has proven valuable for species identification [63], including the detection of hybrid Fasciola species, which had previously been observed using multiplex PCR [64].

In conclusion, from a clinical point of view, these techniques provide exceptional sensitivity and specificity. However, they necessitate intricate methodologies, protocols for nucleic acid extraction, and specialized equipment [65]. This is the reason why these techniques need to be performed in a molecular biology laboratory.

Finally, the Loop-mediated Isothermal Amplification (LAMP) technique has emerged as a suitable alternative to traditional molecular diagnostics. This technique delivers results comparable to PCR, detecting positives at one week post-infection in a shorter time frame. It has proven effective even in stool and water samples [61] and occasionally demonstrates high specificity in fecal samples from sheep and cattle when compared with Fecal Egg Count (FEC) and PCR [66]. In general terms, LAMP appears to be a significant tool in the diagnosis of fasciolosis when compared with on-farm FEC. However, its sensitivity still requires improvement in comparison to FEC [67,68] and ELISA [30].

A summary of these methods is shown in Table 2 and Table 3.

## 3. Treatment

### 3.1. Drug Treatment

Treatment against *F. hepatica* traditionally relied on the use of anthelmintics. Currently, there are seven commercially available compounds that have demonstrated therapeutic activity against *F. hepatica*. These compounds (shown in Table 4), classified chemically, include three benzimidazoles (namely albendazole, ricobendazole, and triclabendazole), one halogenated phenol (nitroxinil), three salicylanilides (closantel, oxyclozanide, and rafoxanide), and one sulphonamide (clorsulon) [69]. However, the efficacy and application of these drugs are subject to various influencing and limiting factors.

The first consideration is that most available drugs are not effective against all stages of *Fasciola* infection [70], which significantly restricts the therapeutic options. In this context, triclabendazole (TCBZ) exhibits high efficacy against both adult parasites and immature flukes as early as 2 days post-infection [71], while other flukicides target flukes from 6 to 14 weeks post-infection [72]. However, the results vary considerably depending on the target ruminant species and dosage. For instance, rafoxanide was moderately to highly effective (86–99%) against 6-week-old flukes in sheep but showed insufficient activity (16–55.7%) in cattle [69]. This is particularly significant in goats due to their higher physiological tolerance, as goats metabolize anthelmintics faster than sheep, especially benzimidazoles [73]. Unfortunately, there are limited studies on the effect of these drugs on fasciolosis in goats.

Another crucial aspect to consider is the established withdrawal periods for treating livestock, particularly dairy livestock. Three periods should be considered in dairy livestock: lactation, dry period, and first gestation. During lactation, only medications with very short withdrawal periods (typically up to 7 days) can be utilized, as the milk from the treated animals is not suitable for human consumption. During the dry period, medications with longer withdrawal periods (up to 60 days) can be administered, as the animals are not producing milk, and the rest interval between successive lactations is traditionally 60 days in cattle. Furthermore, during the first gestation, females are not productive, and therefore, all authorized medications (including those with withdrawal periods of more than 60 days) can be administered [74]. Withdrawal periods for fasciolicides should be harmonized worldwide. Although some international organizations have published guidelines aimed at standardizing such studies, harmonization is challenging due to the multitude of factors influencing drug pharmacokinetics, including breed, animal life stage, age, housing and feeding conditions, and health status [69].

Lastly, it is important to note that EU Member States adhere to a common regulation concerning the usage conditions of veterinary medicinal products and the maximum residue limits of pharmacologically active substances in food derived from animals. However, there are substantial differences between countries and even within the same country in relation to the withdrawal periods associated with some flukicides with similar pharmaceutical formulations. For instance, in the case of triclabendazole, it is noteworthy that no product containing this active substance is marketed for use in cattle in Portugal, Italy, Denmark, or Sweden, and those available in Spain and Belgium are not authorized for dairy livestock [69].

The high efficacy of TCBZ at various stages of *F. hepatica* development has led to its repeated use without rotating with other drugs. This practice, combined with under-dosing and the application of deworming to entire flocks without conducting coprological diagnoses, has inevitably led to TCBZ resistance (TCBZ-R) in liver fluke populations [4,7,75]. Since the first report of TCBZ resistance in Australia in 1995 [76], resistance to TCBZ in *F. hepatica* has been widely reported in many regions of the world, including Europe, South America, and Australia, in both sheep and cattle [7]. In addition to TCBZ, resistance has been reported for other flukicides; for instance, resistance to albendazole has been identified in Argentina [77], Uruguay [78], and Sweden [79,80]. In Sweden, resistance to closantel has also been reported in fluke populations in cattle [81].

Based on the resistance profiles previously commented on, the use of drug combinations is being considered. These combinations can be used to treat mixed infections and have different mechanisms of action, potentially resulting in additive or synergistic effects. This approach could allow for the use of lower drug concentrations and slow down the development of drug resistance [82,83]. For instance, synergism between TCBZ and clorsulon, as well as TCBZ and luxabendazole, at significantly lower dose rates has been demonstrated in 6-week-old infections of TCBZ-resistant *F. hepatica* in sheep. Combinations of closantel, TCBZ, clorsulon, and luxabendazole have been shown to be synergistic against salicylanilide-resistant isolates of *F. hepatica* in sheep of different ages and at reduced dose rates [84]. However, resistance to more than one flukicide in the same fluke population has been reported for combinations of albendazole and TCBZ [85,86], albendazole and clorsulon [24,87], and albendazole, clorsulon, and TCBZ [24].

As we have discussed, the emergence of strains resistant to flukicide drugs necessitates the exploration of alternatives to combat *F. hepatica*. One strategy to enhance efficacy is to increase drug availability by modifying drug metabolism. In this regard, inhibitors of glutathione S-transferase (GST) [88], flavin monooxygenase [89,90,91,92], and cytochrome P450 [93,94] have been shown to potentiate the action of drugs in TCBZ-resistant flukes. Cytochrome P450 has been extensively studied, with miconazole, clotrimazole, and ketoconazole demonstrating the most effective inhibition of this enzyme [95]. For instance, co-administration of TCBZ and ketoconazole has been shown to increase the bioavailability of TCBZ in sheep [96]. Furthermore, the combination of ketoconazole and TCBZ has been demonstrated to potentiate the action of TCBZ against TCBZ-resistant *F. hepatica* [91,92].

Another approach involves the re-purposing of existing or older drugs for potential use as flukicides. For example, tribendimidine, originally developed to treat *Ascaris lumbricoides* infections [97], has been investigated for potential activity against trematode parasites. It has shown activity against *Clonorchis sinensis* and *Opisthorchis viverrini* in a rodent model but not against *F. hepatica* or *Schistosoma mansoni* [9]. Nitazoxanide, which has broad-spectrum activity against intestinal protozoal and helminth infections in humans [98,99], has also been found to be effective in treating human fascioliasis [100,101]. Oxfendazole, typically used to treat nematode and cestode infections, has demonstrated ovicidal activity against TCBZ-S flukes [102] and efficacy against *F. hepatica* infections in sheep at increased dose rates [103].

### 3.2. Alternative Treatment

Recently, and particularly in the last decade, the development of natural compounds derived from plants for disease control and treatment has gained significant traction, including for diseases of parasitic origin. This trend aligns with consumer demand for animal products sourced from organic livestock, which are perceived as natural, healthy, and respectful of animal welfare [69]. Numerous natural products have demonstrated activity against *F. hepatica*, both in vitro and in vivo. Some of these natural products have exhibited in vitro activity against the eggs and miracidia of *F. hepatica*. Recently, a 100% ovicidal effect of *Cuminum cyminum* essential oil has been reported on *F. hepatica* eggs at doses ranging from 0.031125 to 4.15 mg/mL after 14 days of in vitro incubation [104]. Under the same experimental conditions, a 100% ovicidal effect has been reported using concentrations of 0.03375 to 4.5 and 0.031875 to 4.25 mg/mL of the essential oils of *Pelargonium graveolens* and *Citrus aurantium* [105]. Concentrations of 12.5, 25.0, and 50.0 mg/mL of crude extract from *Momordica charantia* L. inhibited miracidia development in 100% of *F. hepatica* eggs after twelve days of in vitro incubation [106]. A complete halt in the development and death of 100% of *F. hepatica* eggs has also been observed at concentrations of 25 and 50 mg/mL of *M. oleifera* seed methanolic extract after 48 and 72 h of in vitro incubation [16].

The anthelmintic efficacy of these natural products has also been demonstrated under in vitro conditions in both the juvenile and adult stages of *F. hepatica*. Five of the fifteen tropical plant extracts screened exhibited in vitro activity against new excysted juveniles (NEJs) [17], and 7-keto-sempervirol, derived from *Lycium chinense*, impacts the movement and viability of NEJs as well as the morphology of adult specimens [107]. Regarding the effect on adult *F. hepatica*, incubation for 24 h with concentrations of 6000 to 8000 µg/mL of *Ferula assa-foetida* hydroalcoholic extract showed 100% mortality in adults [108]. Additionally, the essential oils from *C. cyminum*, *Petroselinum graveolens*, and *Citrus aurantium* caused fluke mortality at concentrations of 0.06225 mg/mL (at 15 h), 0.03375 mg/mL (at 15 h), and 0.06375 mg/mL (at 18 h) of incubation, respectively [104,105].

The demonstration of the in vitro anthelmintic activity of plant extracts is of interest. However, the application of these products to ruminant species requires finding plant-derived compounds with activity against *F. hepatica* under in vivo conditions. Artemisinin derivatives, originally isolated from *Artemisia*, such as artemether and artesunate, have been administered at different doses and routes of administration, showing fasciocidal properties against *F. hepatica* in naturally infected sheep with a significant reduction in egg count (64.9% and 90%, respectively) and significant fluke burden reductions (91.3% and 87.1–91.9%, respectively) with intramuscular administration [8,109]. Reductions of 29.54%, 78.26%, and 82.35% in *F. hepatica* egg counts in naturally infected goats have been reported when the animals were fed a herbal mixture (bio-dewormer) at different dose rates (1000, 1200, and 1400 mg/kg, respectively) for 30 days [110]. In the same way, the use of deoiled mahua seed cake (*Bassia latifolia*) in the diet of buffaloes orally infected with *F. gigantica* metacercariae showed reduced egg count and lower egg output in animals supplemented with this compound [15]. The anthelmintic efficacy of *Moringa oleifera* seed has been tested in rabbits infected with *F. hepatica* (50 metacercariae), showing no flukes recovered with an oral dose of 150 mg/kg body weight [16].

As has been commented on in the preceding paragraphs, the use of alternative plant-derived anthelmintics may be a viable option for treating *Fasciola* infection. However, further studies are needed to implement such treatments, ensuring they can be applied independently rather than in combination with other drugs or dietary supplements.

### 3.3. Treatment in the Future

Although the use of drugs is still the main tool for controlling the infection caused by Fasciola, there are several issues that need to be faced. There are few fasciolicides commercially available, and in some countries in the European region where the prevalence is higher, the drugs available are not effective against early-stage parasites.

Deeper studies need to be performed to enhance the efficacy of existing drugs (through combinations, synergy, etc.) as well as the use of natural compounds that, although promising, still need to be used together with pharmacological drugs or as dietary supplements.

A summary of this section is shown in Table 5.

## 4. Vaccines

As previously discussed, drug resistance against *F. hepatica* has required a shift in treatment strategies over recent years. Various approaches, including the use of alternative anthelmintics, have been explored. Simultaneously, the phenomenon of climate change has led to an increased prevalence of *F. hepatica* worldwide, driven by improved living conditions for both the intermediate host (*Galba truncatula*) and the infective stage (metacercariae) [111,112,113]. Consequently, there is a pressing need to identify a new control method that differs from drug-based approaches. This alternative should be cost-effective, user-friendly, and environmentally friendly, devoid of residues from animal production, and able to provide long-term protection. In this context, the development of an effective vaccine appears to be a suitable and sustainable solution [113,114].

There are only a few veterinary vaccines against livestock helminths available in European territory (Huskvac^®^—against *Dictyocaulus viviparus*—in the UK and Ireland; Barbervax^®^—against *Haemonchus contortus*—in the UK). This serves as an example of the challenge of developing effective vaccines against parasites, particularly helminths. As we write this review, we find that there is still no commercial vaccine available against *F. hepatica*. Over the past years, numerous vaccine trials have been conducted, yielding highly variable results [114,115]. The reasons behind this variability are multifaceted. On one hand, distinct immune responses have been observed across species [116,117], even among different breeds of the same species [114,115]. On the other hand, there remains a lack of clarity regarding both the immune regulatory mechanisms occurring within the host [117,118,119] and the immune evasion strategies employed by the parasite [113,114].

Within the host, *F. hepatica* can induce a Th2 immune response [120,121,122,123,124] while simultaneously down-regulating the Th1 immune response [113,125,126,127]. This strategic manipulation allows the parasite to create its own “favorable immune environment”, ensuring its survival, facilitating its arrival to bile ducts, and mitigating liver damage caused by the host’s immune reaction [113]. Despite these insights, only a subset of the immunoregulatory mechanisms employed by *F. hepatica* are currently understood [128,129,130,131,132]. Consequently, further research funding is essential to delve deeper into this aspect. In the following paragraphs, we will summarize the latest trends observed in various vaccine trials conducted on ruminants.

Initially, the earliest strategies relied on utilizing native *F. hepatica* excretion–secretion (E-S) proteins with antigenic properties [115]. The employment of these native proteins afforded a degree of protection in cattle [120,121,132,133,134], sheep [135,136,137,138,139], and goats [140]. In these experimental studies, protection was primarily assessed by measuring the fluke burden, although fecal egg counts and both gross and microscopic lesions were also evaluated.

The principal challenge in using native proteins was not their acquisition—since, as excretion–secretion products, they are readily obtainable—but rather the substantial number of flukes required to procure a sufficient protein concentration for research purposes. Consequently, once certain proteins were pinpointed as promising candidates, experiments commenced using recombinant versions of these native proteins. These recombinant proteins were simpler to produce in large quantities. Accordingly, further trials were conducted in cattle [141,142,143,144], sheep [11,128,131,137,145,146,147,148,149], and goats [150,151,152,153,154,155]. Although all the assays utilized various adjuvants to enhance the host’s immune response, the outcomes in terms of protection were highly variable. While some studies reported reductions in fluke burden, egg output, or hepatic lesions, only a few demonstrated statistically significant decreases that could be characterized as a definitive level of protection in sheep [11,147,148,156] and goats [151,152,153].

Alternative approaches have been explored by some researchers, such as employing mimotopes of *F. hepatica* in sheep and goats [157,158], yielding promising results in reducing fluke burden and egg outputs. Additionally, the efficacy of DNA vaccines has been investigated [159,160], along with recent trials of nasal [161] and oral vaccines [44], though further research is required to confirm these findings.

It is evident that a significant cohort of researchers worldwide is dedicated to developing an effective vaccine against *F. hepatica*. Because of these efforts, several parasite proteins have been identified as potential vaccine candidates due to their antigenic activity. However, the inconsistent results and varying levels of protection achieved necessitate more in-depth studies and additional vaccine trials to identify the key molecules or molecules capable of conferring immunity against this parasite.

## 5. Conclusions

As a conclusion to this review article, it has been observed that there are various diagnostic methods for fasciolosis, ranging from more direct and easily applicable ones to immunological or biomolecular-based methods that allow for an earlier and more accurate diagnosis. Furthermore, the treatment with pharmaceuticals has been analyzed, identifying the drugs of choice and the existing problem derived from the emergence of resistance, which has led to the development of new treatment protocols and even the search for alternative treatments. Finally, the control of the disease through the search for an effective vaccine has been addressed, with different strategies and approaches followed by various researchers. Although immunological control of the disease is a viable option, more studies are still needed in this regard, as well as a deeper understanding of the immunological mechanisms established in the parasite–host interaction.

## Figures and Tables

**Table 1:** Summary of the different types of direct methods (and characteristics) for the diagnosis of *F. hepatica*.

Direct Methods		
Necropsy	Fecal Egg Count	
Not antemortem	Antemortem	
Accurate diagnosis of infection (fluke burden)	Sedimentation	Flotation
	Flukefinder^®^ *	FLOTAC^®^/Mini-FLOTAC^®^ *

* Higher sensitivity than traditional methods for egg burden >20 epg.

**Table 2:** Summary of the different indirect methods (and characteristics) based on immunodiagnosis for the diagnosis of *F. hepatica*.

Indirect Methods	
ELISA	
**Indirect**	**Sandwich**
Early detection (2–4 weeks)	Detect active infections
No kits available	Commercial kits available
Used in serum samples	Used in serum, milk, and fecal samples
Cross reaction (some antigens)	
**Advantages**	**Advantages**
High sensitivity	High sensitivity
Early detection	Early detection
**Disadvantages**	**Disadvantages**
Economical costs	Economical costs
False positives/negatives	False positives/negatives
Specific equipment	Specific equipment

**Table 3:** Summary of the different indirect methods (and characteristics) based on molecular techniques for the diagnosis of *F. hepatica*.

Indirect Methods			
Molecular Techniques			
**PCR**	**RT-PCR**	**PCR-RFLP**	**LAMP**
**Advantages**			
Species differentiation			
High sensitivity High specificity			
			one-week detection different samples (stool, water)
**Disadvantages**			
Specific equipment Complex protocols DNA source (parasite eggs)			

**Table 4:** Drugs used against *F. hepatica*.

Group	Chemical Name	Use	Efficacy
Benzimidazoles	Albendazole	Oral	Adults
	Ricobendazole	Oral	Adults
	Triclabendazole	Oral	Adults and immature (2 days of age)
Halogenated phenol	Nitroxinil		
Salicylanilides	Closantel	Oral	Adults and immature (5 weeks of age)
	Oxyclozanide	Oral	Adults
	Rafoxanide	Oral	Adults
Sulphonamide	Clorsulon	Oral	Adults

**Table 5:** Summary of the different treatments (and characteristics) against *F. hepatica*.

Treatment	
Drug Treatment	Alternative Treatment
7 compounds Not effective in all stages (except TCBZ) Withdrawal periods	Natural products derived from plants Compatible with organic livestock Respectful of animal welfare
**Drugs combinations**	**Plants/Stage target**
TCBZ + clorsulon TCBZ + luxabendazole	*C. cyminun*/eggs + adults *P. graveolens*/eggs + adults *C. aurantium*/eggs + adults *Artemisia*/eggs + adults *B. latifolia*/eggs *M. charantia*/miracidium *M. oleifera*/miracidium + adults *L. chinense*/NEJs + adults *F. assa-foetida*/adults
**Re-purposing older drugs**	
Tribendimidine Nitrazoxanide Oxfendazole	
**Increasing drug metabolism**	
Cytochrome P450 TCBZ + ketoconazole	
**Disadvantages**	**Disadvantages**
Not effective in all stages (except TCBZ) Withdrawal periods Resistance phenomena	Promising (but further studies needed) Combination with other drugs Used as dietary supplements

## References

[1] Webb C.M., Cabada M.M. (2018). Recent developments in the epidemiology, diagnosis, and treatment of *Fasciola* infection. Curr. Opin. Infect. Dis..

[2] Mehmood K., Zhang H., Sabir A.J., Abbas R.Z., Ijaz M., Durrani A.Z., Saleem M.H., Ur Rehman M., Iqbal M.K., Wang Y. (2017). A review on epidemiology, global prevalence and economical losses of fasciolosis in ruminants. Microb. Pathog..

[3] Dirección de Producción y Sanidad Animal de la FAO (2003). Resistencia a los Antiparasitarios. Estado Actual con Énfasis en América Latina.

[4] Beesley N.J., Caminade C., Charlier J., Flynn R.J., Hodgkinson J.E., Martinez-Moreno A., Martinez-Valladares M., Perez J., Rinaldi L., Williams D.J.L. (2018). *Fasciola* and fasciolosis in ruminants in Europe: Identifying research needs. Transbound. Emerg. Dis..

[5] Mas-Coma S., Valero M.A., Bargues M.D. (2023). One Health for fascioliasis control in human endemic areas. Trends Parasitol..

[6] Rehman T., Elsaid F.G., Garijo Toledo M.M., Gentile A., Gul R.A., Rashid M., Aleem M.T., Zaman M.A. (2023). Fasciolosis: Recent Update in Vaccines Development and Their Efficacy. Pak. Vet. J..

[7] Fairweather I., Brennan G.P., Hanna R.E.B., Robinson M.W., Skuce P.J. (2020). Drug resistance in liver flukes. Int. J. Parasitol. Drugs Drug Resist..

[8] Keiser J., Rinaldi L., Veneziano V., Mezzino L., Tanner M., Utzinger J., Cringoli G. (2008). Efficacy and safety of artemether against a natural *Fasciola hepatica* infection in sheep. Parasitol. Res..

[9] Keiser J., Utzinger J. (2007). Advances in the discovery and development of trematocidal drugs. Expert Opin. Drug Discov..

[10] Rioux M.-C., Carmona C., Acosta D., Ward B., Ndao M., Gibbs B.F., Bennett H.P., Spithill T.W. (2008). Discovery and validation of serum biomarkers expressed over the first twelve weeks of *Fasciola hepatica* infection in sheep. Int. J. Parasitol..

[11] Zafra R., Buffoni L., Pérez-Caballero R., Molina-Hernández V., Ruiz-Campillo M.T., Pérez J., Martínez-Moreno Á., Martínez Moreno F.J. (2021). Efficacy of a multivalent vaccine against *Fasciola hepatica* infection in sheep. Vet. Res..

[12] Flores-Ramos M., Leyva-Gómez G., Rojas-Campos T., Cruz-Mendoza I., Hernández-Campos A., Vera-Montenegro Y., Castillo R., Velázquez-Martínez I., Padierna-Mota C., Arias-García R. (2024). Fosfatriclaben, a prodrug of triclabendazole: Preparation, stability, and fasciolicidal activity of three new intramuscular formulations. Vet. Parasitol..

[13] Talaie H., Emami H., Yadegarinia D., Nava-Ocampo A.A., Massoud J., Azmoudeh M., Mas-Coma S. (2004). Randomized trial of a single, double and triple dose of 10 mg/kg of a human formulation of Triclabendazol in patients with Fascioliasis. Clin. Exp. Pharmacol. Physiol..

[14] Beesley N.J., Williams D.J.L., Paterson S., Hodgkinson J. (2017). *Fasciola hepatica* demonstrates high levels of genetic diversity, a lack of population structure and high gene flow: Possible implications for drug resistance. Int. J. Parasitol..

[15] Singh P., Verma A.K., Jacob A.B., Gupta S.C., Mehra U.R. (2011). Haematological and biochemical changes in *Fasciola gigantica* infected buffaloes fed on diet containing deoiled mahua (*Bassia latifolia*) seed cake. J. Appl. Anim. Res..

[16] Kandil O.M., Hassan N.M.F., Sedky D., Ata E.B., Nassar S.A., Shalaby H.A., Nanev V., Tsocheva-Gaytandzhieva N., Gabrashanska M. (2018). Anthelmintic efficacy of *Moringa oleifera* seed methanolic extract against *Fasciola hepatica*. J. Parasit. Dis..

[17] Alvarez-Mercado J.M., Ibarra-Velarde F., Alonso-Díaz M.Á., Vera-Montenegro Y., Avila-Acevedo J.G., García-Bores A.M. (2015). In vitro antihelmintic effect of fifteen tropical plant extracts on excysted flukes of *Fasciola hepatica*. BMC Vet. Res..

[18] Mazeri S., Sargison N., Kelly R.F., de C. Bronsvoort B.M., Handel I. (2016). Evaluation of the Performance of Five Diagnostic Tests for *Fasciola hepatica* Infection in Naturally Infected Cattle Using a Bayesian No Gold Standard Approach. PLoS ONE.

[19] Cringoli G., Rinaldi L., Maurelli M.P., Utzinger J. (2010). FLOTAC: New multivalent techniques for qualitative and quantitative copromicroscopic diagnosis of parasites in animals and humans. Nat. Protoc..

[20] Foreyt W.J. (2001). Veterinary Parasitology. Reference Manual.

[21] Salimi-Bejestani M.R., McGarry J.W., Felstead S., Ortiz P., Akca A., Williams D.J.L. (2005). Development of an antibody-detection ELISA for *Fasciola hepatica* and its evaluation against a commercially available test. Res. Vet. Sci..

[22] Martínez-Pérez J.M., Robles-Pérez D., Rojo-Vázquez F.A., Martínez-Valladares M. (2014). Immunological features of LPS from Ochrobactrum intermedium on sheep experimentally infected with *Fasciola hepatica*. Res. Vet. Sci..

[23] Martínez-Pérez J.M., Robles-Pérez D., Rojo-Vázquez F.A., Martínez-Valladares M. (2012). Comparison of three different techniques to diagnose *Fasciola hepatica* infection in experimentally and naturally infected sheep. Vet. Parasitol..

[24] Robles-Pérez D., Martínez-Pérez J.M., Rojo-Vázquez F.A., Martínez-Valladares M. (2013). The diagnosis of fasciolosis in feces of sheep by means of a PCR and its application in the detection of anthelmintic resistance in sheep flocks naturally infected. Vet. Parasitol..

[25] Le T.H., Nguyen K.T., Nguyen N.T.B., Doan H.T.T., Le X.T.K., Hoang C.T.M., De N.V. (2012). Development and Evaluation of a Single-Step Duplex PCR for Simultaneous Detection of *Fasciola hepatica* and *Fasciola gigantica* (Family Fasciolidae, Class Trematoda, Phylum Platyhelminthes). J. Clin. Microbiol..

[26] Corrales J.L., McEvoy A., Lalor R., Cwiklinski K., Doyle S., Parkinson M., Keane O.M., Dalton J.P., Dorey A.L. (2023). The use of cathepsin L1 (FhCL1) serological ELISA in sentinel screening for liver fluke on sheep farms. Vet. Parasitol. Reg. Stud. Rep..

[27] Sargison N.D., Scott P.R. (2011). Diagnosis and economic consequences of triclabendazole resistance in *Fasciola hepatica* in a sheep flock in south-east Scotland. Vet. Rec..

[28] Paras K.L., George M.M., Vidyashankar A.N., Kaplan R.M. (2018). Comparison of fecal egg counting methods in four livestock species. Vet. Parasitol..

[29] Brockwell Y.M., Spithill T.W., Anderson G.R., Grillo V., Sangster N.C. (2013). Comparative kinetics of serological and coproantigen ELISA and faecal egg count in cattle experimentally infected with *Fasciola hepatica* and following treatment with triclabendazole. Vet. Parasitol..

[30] Arifin M.I., Höglund J., Novobilský A. (2016). Comparison of molecular and conventional methods for the diagnosis of *Fasciola hepatica* infection in the field. Vet. Parasitol..

[31] Charlier J., De Meulemeester L., Claerebout E., Williams D., Vercruysse J. (2008). Qualitative and quantitative evaluation of coprological and serological techniques for the diagnosis of fasciolosis in cattle. Vet. Parasitol..

[32] Becker A.-C., Kraemer A., Epe C., Strube C. (2016). Sensitivity and efficiency of selected coproscopical methods—Sedimentation, combined zinc sulfate sedimentation-flotation, and McMaster method. Parasitol. Res..

[33] Kurnianto H., Ramanoon S.Z., Aziz N.A.A., Indarjulianto S. (2022). Prevalence, risk factors, and infection intensity of fasciolosis in dairy cattle in Boyolali, Indonesia. Vet. World.

[34] Zárate-Rendón D.A., Vlaminck J., Levecke B., Briones-Montero A., Geldhof P. (2019). Comparison of Kato-Katz Thick Smear, Mini-FLOTAC, and Flukefinder for the Detection and Quantification of *Fasciola hepatica* Eggs in Artificially Spiked Human Stool. Am. J. Trop. Med. Hyg..

[35] Kahl A., von Samson-Himmelstjerna G., Helm C.S., Hodgkinson J., Williams D., Weiher W., Terhalle W., Steuber S., Krücken J. (2023). Coproscopical diagnosis of patent *Fasciola hepatica* infections in sheep—A comparison between standard sedimentation, FLUKEFINDER® and a combination of both. Vet. Parasitol..

[36] Bosco A., Ciuca L., Maurelli M.P., Vitiello P., Cringoli G., Prada J.M., Rinaldi L. (2023). Comparison of Mini-FLOTAC, Flukefinder® and sedimentation techniques for detection and quantification of *Fasciola hepatica* and Calicophoron daubneyi eggs using spiked and naturally infected bovine faecal samples. Parasit. Vectors.

[37] Nur Hafizah S., Noor Izani N.J., Ahmad Najib M., Wan-Nor-Amilah W.A.W. (2023). Immunodiagnosis of Fascioliasis in Ruminants by ELISA Method: A Mini-Review. Malays. J. Med. Sci. MJMS.

[38] Alvarez Rojas C.A., Jex A.R., Gasser R.B., Scheerlinck J.-P.Y., Rollinson D., Stothard J.R. (2014). Techniques for the Diagnosis of *Fasciola* Infections in Animals: Room for Improvement. Advances in Parasitology.

[39] Awad W.S., Ibrahim A.K., Salib F.A. (2009). Using indirect ELISA to assess different antigens for the serodiagnosis of *Fasciola gigantica* infection in cattle, sheep and donkeys. Res. Vet. Sci..

[40] Martínez-Sernández V., Perteguer M.J., Hernández-González A., Mezo M., González-Warleta M., Orbegozo-Medina R.A., Romarís F., Paniagua E., Gárate T., Ubeira F.M. (2018). Comparison of recombinant cathepsins L1, L2, and L5 as ELISA targets for serodiagnosis of bovine and ovine fascioliasis. Parasitol. Res..

[41] Cwiklinski K., Dalton J.P. (2018). Advances in *Fasciola hepatica* research using ‘omics’ technologies. Int. J. Parasitol..

[42] Cwiklinski K., Robinson M.W., Donnelly S., Dalton J.P. (2021). Complementary transcriptomic and proteomic analyses reveal the cellular and molecular processes that drive growth and development of *Fasciola hepatica* in the host liver. BMC Genom..

[43] Wesołowska A., Basałaj K., Norbury L.J., Sielicka A., Wędrychowicz H., Zawistowska-Deniziak A. (2018). Vaccination against *Fasciola hepatica* using cathepsin L3 and B3 proteases delivered alone or in combination. Vet. Parasitol..

[44] López Corrales J., Cwiklinski K., De Marco Verissimo C., Dorey A., Lalor R., Jewhurst H., McEvoy A., Diskin M., Duffy C., Cosby S.L. (2021). Diagnosis of sheep fasciolosis caused by *Fasciola hepatica* using cathepsin L enzyme-linked immunosorbent assays (ELISA). Vet. Parasitol..

[45] Jacob S.S., Sengupta P.P., Pavithra B.S., Chandu A.G.S., Raina O.K. (2023). Development of an enzyme linked immunosorbent assay using recombinant cathepsin B5 antigen for sero-surveillance of bovine tropical fasciolosis. Vet. Parasitol..

[46] Gong J.-Z., Fan Y.-M., Yuan W., Pan M., Liu D., Tao J.-P., Huang S.-Y. (2023). Development of a novel method for diagnosis of fasciolosis based on cathepsin L7 in ruminants. Vet. Parasitol..

[47] Ahumada M., Godino A., Guasconi L., Deheza C., Amaranto M., Pruzzo C.I., Vitulli-Moya G., Chiapello L., Carrizo M.E., Barra J.L. (2024). Antibody detection against Kunitz-type protein in *Fasciola hepatica* experimentally infected sheep using enzyme-linked immunosorbent assay (ELISA). Int. J. Vet. Sci. Med..

[48] Anuracpreeda P., Chawengkirttikul R., Sobhon P. (2016). Immunodiagnosis of *Fasciola gigantica* Infection Using Monoclonal Antibody-Based Sandwich ELISA and Immunochromatographic Assay for Detection of Circulating Cathepsin L1 Protease. PLoS ONE.

[49] Mokhtarian K., Akhlaghi L., Mohammadi M., Meamar A.R., Razmjou E., Khoshmirsafa M., Falak R. (2016). Evaluation of anti-Cathepsin L1: A more reliable method for serodiagnosis of human fasciolosis. Trans. R. Soc. Trop. Med. Hyg..

[50] Mokhtarian K., Akhlaghi L., Meamar A.R., Razmjou E., Manouchehri Naeini K., Gholami S., Najafi Samei M., Falak R. (2016). Serodiagnosis of fasciolosis by fast protein liquid chromatography-fractionated excretory/secretory antigens. Parasitol. Res..

[51] Mokhtarian K., Meamar A.R., Khoshmirsafa M., Razmjou E., Masoori L., Khanmohammadi M., Akhlaghi L., Falak R. (2018). Comparative assessment of recombinant and native immunogenic forms of *Fasciola hepatica* proteins for serodiagnosis of sheep fasciolosis. Parasitol. Res..

[52] Kuerpick B., Schnieder T., Strube C. (2013). Evaluation of a recombinant cathepsin L1 ELISA and comparison with the Pourquier and ES ELISA for the detection of antibodies against *Fasciola hepatica*. Vet. Parasitol..

[53] Kelly R.F., Mazeri S., Hartley C., Hamman S.M., Ngu Ngwa V., Nkongho E.F., Tanya V., Sander M., Ndip L., Morgan K.L. (2019). Assessing the performance of a *Fasciola gigantica* serum antibody ELISA to estimate prevalence in cattle in Cameroon. BMC Vet. Res..

[54] Mezo M., González–Warleta M., Castro-Hermida J.A., Carro C., Ubeira F.M. (2010). Kinetics of anti-*Fasciola* IgG antibodies in serum and milk from dairy cows during lactation, and in serum from calves after feeding colostrum from infected dams. Vet. Parasitol..

[55] Mezo M., González-Warleta M., Ubeira F.M. (2007). The use of MM3 monoclonal antibodies for the early immunodiagnosis of ovine fascioliasis. J. Parasitol..

[56] Mezo M., González-Warleta M., Carro C., Ubeira F.M. (2004). An ultrasensitive capture elisa for detection of *Fasciola hepatica* coproantigens in sheep and cattle using a new monoclonal antibody (MM3). J. Parasitol..

[57] Martínez-Sernández V., Orbegozo-Medina R.A., González-Warleta M., Mezo M., Ubeira F.M. (2016). Rapid Enhanced MM3-COPRO ELISA for Detection of *Fasciola* Coproantigens. PLoS Negl. Trop. Dis..

[58] Mezo M., González-Warleta M., Castro-Hermida J.A., Martínez-Sernández V., Ubeira F.M. (2022). Field evaluation of the enhanced MM3-COPRO ELISA test for the diagnosis of *Fasciola hepatica* infection in sheep. PLoS ONE.

[59] Anuracpreeda P., Chawengkirtikul R., Tinikul Y., Poljaroen J., Chotwiwatthanakun C., Sobhon P. (2013). Diagnosis of *Fasciola gigantica* infection using a monoclonal antibody-based sandwich ELISA for detection of circulating cathepsin B3 protease. Acta Trop..

[60] Munita M.P., Rea R., Martinez-Ibeas A.M., Byrne N., Kennedy A., Sekiya M., Mulcahy G., Sayers R. (2019). Comparison of four commercially available ELISA kits for diagnosis of *Fasciola hepatica* in Irish cattle. BMC Vet. Res..

[61] Tran L., Toet H., Beddoe T. (2022). Environmental detection of *Fasciola hepatica* by loop-mediated isothermal amplification. PeerJ.

[62] Calvani N.E.D., Windsor P.A., Bush R.D., Šlapeta J. (2017). Scrambled eggs: A highly sensitive molecular diagnostic workflow for *Fasciola* species specific detection from faecal samples. PLoS Negl. Trop Dis..

[63] Heydarian P., Javadi Mamaghani A., Hajialilo E., Bozorgomid A., Mohaghegh M., Aryaeipour M., Abbaszadeh Afshar M.J., Jajarmi V. (2024). Identification and differentiation of *Fasciola hepatica* and *F. gigantica* using multiplex PCR technique. Ann. Parasitol..

[64] Sharbatkhori M., Nasibi S., Mohammadi M.A., Aryaeipour M., Raeghi S., Fasihi Harandi M. (2023). Morphological and molecular characterization of *Fasciola* isolates from livestock in Golestan province, northern Iran. Vet. Med. Sci..

[65] Uzun V., Celik F., Simsek S., Kesik H.K., Kilinc S.G., Zhang X., Ahmed H., Cao J. (2022). Multiplex PCR and Sequence Analysis to Investigate Genetic Diversity of *Fasciola* Isolates from Cattle and Sheep in Turkey. Pathogens.

[66] Lee P.L.M. (2017). DNA amplification in the field: Move over PCR, here comes LAMP. Mol. Ecol. Resour..

[67] Amiri S., Shemshadi B., Shirali S., Kheirandish F., Fallahi S. (2021). Accurate and rapid detection of *Fasciola hepatica* copro-DNA in sheep using loop-mediated isothermal amplification (LAMP) technique. Vet. Med. Sci..

[68] Bari T., Al-Mamun M.A., Toet H., Rathinasamy V., Larkins J.-A., Beddoe T., Spithill T.W., Piedrafita D., Greenhill A. (2023). Evaluation of Lamp for *Fasciola* Hepatica Detection from Faecal Samples of Experimentally and Naturally Infected Cattle. Vet. Parasitol..

[69] Castro-Hermida J.A., González-Warleta M., Martínez-Sernández V., Ubeira F.M., Mezo M. (2021). Current Challenges for Fasciolicide Treatment in Ruminant Livestock. Trends Parasitol..

[70] George S.D., Baker K., Lake L., Vanhoff K., D’Arcy R., Emery D., Rolfe P.F. (2017). Characterization of multiple life stages of two Australian *Fasciola hepatica* isolates in sheep. Vet. Parasitol..

[71] Boray J.C., Crowfoot P., Strong M., Allison J., Schellenbaum M., von Orelli M., Sarasin G. (1983). Treatment of Immature and Mature *Fasciola hepatica* infections in Sheep with Triclabendazole. Vet. Rec..

[72] Kelley J.M., Elliott T.P., Beddoe T., Anderson G., Skuce P., Spithill T.W. (2016). Current Threat of Triclabendazole Resistance in *Fasciola hepatica*. Trends Parasitol..

[73] Domke A.V., Chartier C., Gjerde B., Leine N., Vatn S., Østerås O., Stuen S. (2011). Worm control practice against gastro-intestinal parasites in Norwegian sheep and goat flocks. Acta Vet. Scand..

[74] Zobel G., Weary D.M., Leslie K.E., Von Keyserlingk M.A.G. (2015). Invited review: Cessation of lactation: Effects on animal welfare. J. Dairy Sci..

[75] Overend D., Bowen F. (1995). Resistance of *Fasciola hepatica* to triclabendazole. Aust. Vet. J..

[76] Sanabria R., Ceballos L., Moreno L., Romero J., Lanusse C., Alvarez L. (2013). Identification of a field isolate of *Fasciola hepatica* resistant to albendazole and susceptible to triclabendazole. Vet. Parasitol..

[77] Ceballos L., Canton C., Pruzzo C., Sanabria R., Moreno L., Sanchis J., Suarez G., Ortiz P., Fairweather I., Lanusse C. (2019). The egg hatch test: A useful tool for albendazole resistance diagnosis in *Fasciola hepatica*. Vet. Parasitol..

[78] Novobilský A., Averpil H.B., Höglund J. (2012). The field evaluation of albendazole and triclabendazole efficacy against *Fasciola hepatica* by coproantigen ELISA in naturally infected sheep. Vet. Parasitol..

[79] Novobilský A., Amaya Solis N., Skarin M., Höglund J. (2016). Assessment of flukicide efficacy against *Fasciola hepatica* in sheep in Sweden in the absence of a standardised test. Int. J. Parasitol. Drugs Drug Resist..

[80] Novobilský A., Höglund J. (2015). First report of closantel treatment failure against *Fasciola hepatica* in cattle. Int. J. Parasitol. Drugs Drug Resist..

[81] Bartram D.J., Leathwick D.M., Taylor M.A., Geurden T., Maeder S.J. (2012). The role of combination anthelmintic formulations in the sustainable control of sheep nematodes. Vet. Parasitol..

[82] Geary T., Hosking B.C., Skuce P.J., Von Samson-Himmelstjerna G., Maeder S., Holdsworth P., Pomroy W.E., Vercruysse J. (2012). World Association for the Advancement of Veterinary Parasitology (W.A.A.V.P.) guideline: Anthelmintic combination products targeting nematode infections of ruminants and horses. Vet. Parasitol..

[83] Fairweather I., Boray J.C. (1999). Fasciolicides: Efficacy, Actions, Resistance and its Management. Vet. J..

[84] Martínez-Valladares M., Del Rosario Famularo M., Fernández-Pato N., Castañón-Ordóñez L., Cordero-Pérez C., Rojo-Vázquez F.A. (2010). Efficacy of nitroxynil against *Fasciola hepatica* resistant to triclabendazole in a naturally infected sheep flock. Parasitol. Res..

[85] Robles-Pérez D., Martínez-Pérez J.M., Rojo-Vázquez F.A., Martínez-Valladares M. (2015). Screening anthelmintic resistance to triclabendazole in *Fasciola hepatica* isolated from sheep by means of an egg hatch assay. BMC Vet. Res..

[86] Robles-Pérez D., Martínez-Pérez J.M., Rojo-Vázquez F.A., Martínez-Valladares M. (2014). Development of an egg hatch assay for the detection of anthelmintic resistance to albendazole in *Fasciola hepatica* isolated from sheep. Vet. Parasitol..

[87] Fernández V., Estein S., Ortiz P., Luchessi P., Solana V., Solana H. (2015). A single amino acid substitution in isozyme GST mu in Triclabendazole resistant *Fasciola hepatica* (Sligo strain) can substantially influence the manifestation of anthelmintic resistance. Exp. Parasitol..

[88] Devine C., Brennan G.P., Lanusse C.E., Alvarez L.I., Trudgett A., Hoey E., Fairweather I. (2009). Effect of the metabolic inhibitor, methimazole on the drug susceptibility of a triclabendazole-resistant isolate of *Fasciola hepatica*. Parasitology.

[89] Devine C., Brennan G.P., Lanusse C.E., Alvarez L.I., Trudgett A., Hoey E., Fairweather I. (2010). Potentiation of triclabendazole sulphoxide-induced tegumental disruption by methimazole in a triclabendazole-resistant isolate of *Fasciola hepatica*. Parasitol. Res..

[90] Devine C., Brennan G.P., Lanusse C.E., Alvarez L.I., Trudgett A., Hoey E., Fairweather I. (2011). Enhancement of triclabendazole action in vivo against a triclabendazole-resistant isolate of *Fasciola hepatica* by co-treatment with ketoconazole. Vet. Parasitol..

[91] Devine C., Brennan G.P., Lanusse C.E., Alvarez L.I., Trudgett A., Hoey E., Fairweather I. (2012). Potentiation of triclabendazole action in vivo against a triclabendazole-resistant isolate of *Fasciola hepatica* following its co-administration with the metabolic inhibitor, ketoconazole. Vet. Parasitol..

[92] Pakharukova M.Y., Ershov N.I., Vorontsova E.V., Katokhin A.V., Merkulova T.I., Mordvinov V.A. (2012). Cytochrome P450 in fluke Opisthorchis felineus: Identification and characterization. Mol. Biochem. Parasitol..

[93] Pakharukova M.Y., Vavilin V.A., Sripa B., Laha T., Brindley P.J., Mordvinov V.A. (2015). Functional Analysis of the Unique Cytochrome P450 of the Liver Fluke Opisthorchis felineus. PLoS Negl. Trop. Dis..

[94] Mordvinov V.A., Shilov A.G., Pakharukova M.Y. (2017). Anthelmintic activity of cytochrome P450 inhibitors miconazole and clotrimazole: In-Vitro effect on the liver fluke Opisthorchis felineus. Int. J. Antimicrob. Agents.

[95] Virkel G., Lifschitz A., Sallovitz J., Ballent M., Scarcella S., Lanusse C. (2009). Inhibition of cytochrome P450 activity enhances the systemic availability of triclabendazole metabolites in sheep. J. Vet. Pharmacol. Ther..

[96] Xiao S.-H., Utzinger J., Tanner M., Keiser J., Xue J. (2013). Advances with the Chinese anthelminthic drug tribendimidine in clinical trials and laboratory investigations. Acta Trop..

[97] Gilles H.M., Hoffman P.S. (2002). Treatment of intestinal parasitic infections: A review of nitazoxanide. Trends Parasitol..

[98] Hemphill A., Mueller J., Esposito M. (2006). Nitazoxanide, a broad-spectrum thiazolide anti-infective agent for the treatment of gastrointestinal infections. Expert Opin. Pharmacother..

[99] Zumaquero-Ríos J.L., Sarracent-Pérez J., Rojas-García R., Rojas-Rivero L., Martínez-Tovilla Y., Valero M.A., Mas-Coma S. (2013). Fascioliasis and Intestinal Parasitoses Affecting Schoolchildren in Atlixco, Puebla State, Mexico: Epidemiology and Treatment with Nitazoxanide. PLoS Negl. Trop. Dis..

[100] Ramadan H.K.-A., Hassan W.A., Elossily N.A., Ahmad A.A., Mohamed A.A., Abd-Elkader A.S., Abdelsalam E.M.N., Khojah H.M.J. (2019). Evaluation of nitazoxanide treatment following triclabendazole failure in an outbreak of human fascioliasis in Upper Egypt. PLoS Negl. Trop. Dis..

[101] Alvarez L., Moreno G., Moreno L., Ceballos L., Shaw L., Fairweather I., Lanusse C. (2009). Comparative assessment of albendazole and triclabendazole ovicidal activity on *Fasciola hepatica* eggs. Vet. Parasitol..

[102] Lopez-Urbina M.T., Garcia H.H., Gonzalez A.E., Gomez-Puerta L.A., Gavidia C., For The Cysticercosis Working Group in Peru (2012). Efficacy of a Single Oral Dose of Oxfendazole against *Fasciola hepatica* in Naturally Infected Sheep. Am. J. Trop. Med. Hyg..

[103] Brauner De Mello A., Baccega B., Obelar Martins F., Ignês De Santi I., Islabão Y.W., De Giacometi M., Pereira Soares M., Da Rosa Farias N.A., Belmonte Oliveira C. (2023). Activity of cumin essential oil to control fascioliasis: Efficacy and changes in the tegument of *Fasciola hepatica*. Exp. Parasitol..

[104] De Mello A.B., Baccega B.F., Martins F.O., Da Rosa Farias N.A., De Giacometi M., Da Fonseca R.N., De Oliveira Hübner S., Soares M.P., Oliveira C.B. (2023). Microscopic alterations in *Fasciola hepatica* treated with the essential oils of Pelargonium graveolens and Citrus aurantium. Vet. Parasitol..

[105] Pereira C.A.J., Oliveira L.L.S., Coaglio A.L., Santos F.S.O., Cezar R.S.M., Mendes T., Oliveira F.L.P., Conzensa G., Lima W.S. (2016). Anti-helminthic activity of Momordica charantia L. against *Fasciola hepatica* eggs after twelve days of incubation in vitro. Vet. Parasitol..

[106] Edwards J., Brown M., Peak E., Bartholomew B., Nash R.J., Hoffmann K.F. (2015). The Diterpenoid 7-Keto-Sempervirol, Derived from Lycium chinense, Displays Anthelmintic Activity against both Schistosoma mansoni and *Fasciola hepatica*. PLoS Negl. Trop. Dis..

[107] Arbabi M., Haddad A., Esmaeli M., Hooshyar H., Sehat M. (2023). In Vitro Anthelmintic Effect of Ferula assa-foetida Hydroalcoholic Extract Against Flukes of *Fasciola hepatica* and Dicrocoelium dendriticum. Jundishapur J. Nat. Pharm. Prod..

[108] Keiser J., Veneziano V., Rinaldi L., Mezzino L., Duthaler U., Cringoli G. (2010). Anthelmintic activity of artesunate against *Fasciola hepatica* in naturally infected sheep. Res. Vet. Sci..

[109] Abbas R.Z. (2020). Anthelmintic Effects and Toxicity Analysis of Herbal Dewormer against the Infection of Haemonchus contortus and *Fasciola hepatica* in Goat. Pak. Vet. J..

[110] Piedrafita D., Spithill T.W., Raadsma H.W. (2010). Improving animal and human health through understanding liver fluke immunology. Parasite Immunol..

[111] Charlier J., Vercruysse J., Morgan E.R., Van Dijk J., Williams D.J. (2014). Recent advances in the diagnosis, impact on production and prediction of *Fasciola hepatica* in cattle. Parasitology.

[112] Molina-Hernández V., Mulcahy G., Pérez J., Martínez-Moreno Á., Donnelly S., O’Neill S.M., Dalton J.P., Cwiklinski K. (2015). *Fasciola hepatica* vaccine: We may not be there yet but we’re on the right road. Vet. Parasitol..

[113] McManus D.P. (2020). Recent Progress in the Development of Liver Fluke and Blood Fluke Vaccines. Vaccines.

[114] Toet H., Piedrafita D.M., Spithill T.W. (2014). Liver fluke vaccines in ruminants: Strategies, progress and future opportunities. Int. J. Parasitol..

[115] Dominguez M.F., González-Miguel J., Carmona C., Dalton J.P., Cwiklinski K., Tort J., Siles-Lucas M. (2018). Low allelic diversity in vaccine candidates genes from different locations sustain hope for *Fasciola hepatica* immunization. Vet. Parasitol..

[116] Zhang J., Sun Y., Zheng J. (2021). Prospects for liver fluke vaccines. Exp. Parasitol..

[117] Lalrinkima H., Lalchhandama C., Jacob S.S., Raina O.K., Lallianchhunga M.C. (2021). Fasciolosis in India: An overview. Exp. Parasitol..

[118] Siles-Lucas M., Becerro-Recio D., Serrat J., González-Miguel J. (2021). Fascioliasis and fasciolopsiasis: Current knowledge and future trends. Res. Vet. Sci..

[119] Mulcahy G., O’Connor F., McGonigle S., Dowd A., Clery D.G., Andrews S.J., Dalton J.P. (1998). Correlation of specific antibody titre and avidity with protection in cattle immunized against *Fasciola hepatica*. Vaccine.

[120] Mulcahy G., O’Connor F., Clery D., Hogan S.F., Dowd A.J., Andrews S.J., Dalton J.P. (1999). Immune responses of cattle to experimental anti-*Fasciola hepatica* vaccines. Res. Vet. Sci..

[121] Walsh K.P., Brady M.T., Finlay C.M., Boon L., Mills K.H.G. (2009). Infection with a helminth parasite attenuates autoimmunity through TGF-beta-mediated suppression of Th17 and Th1 responses. J. Immunol..

[122] Donnelly S., O’Neill S.M., Sekiya M., Mulcahy G., Dalton J.P. (2005). Thioredoxin peroxidase secreted by *Fasciola hepatica* induces the alternative activation of macrophages. Infect. Immun..

[123] Donnelly S., Stack C.M., O’Neill S.M., Sayed A.A., Williams D.L., Dalton J.P. (2008). Helminth 2-Cys peroxiredoxin drives Th2 responses through a mechanism involving alternatively activated macrophages. FASEB J. Off. Publ. Fed. Am. Soc. Exp. Biol..

[124] Brady M.T., O’Neill S.M., Dalton J.P., Mills K.H. (1999). *Fasciola hepatica* suppresses a protective Th1 response against Bordetella pertussis. Infect. Immun..

[125] O’Neill S.M., Brady M.T., Callanan J.J., Mulcahy G., Joyce P., Mills K.H., Dalton J.P. (2000). *Fasciola hepatica* infection downregulates Th1 responses in mice. Parasite Immunol..

[126] Flynn R.J., Mulcahy G., Welsh M., Cassidy J.P., Corbett D., Milligan C., Andersen P., Strain S., McNair J. (2009). Co-Infection of cattle with *Fasciola hepatica* and Mycobacterium bovis- immunological consequences. Transbound. Emerg. Dis..

[127] Pérez-Caballero R., Siles-Lucas M., González-Miguel J., Martínez-Moreno F.J., Escamilla A., Pérez J., Martínez-Moreno A., Buffoni L. (2018). Pathological, immunological and parasitological study of sheep vaccinated with the recombinant protein 14-3-3z and experimentally infected with *Fasciola hepatica*. Vet. Immunol. Immunopathol..

[128] Ruiz-Campillo M.T., Molina-Hernández V., Pérez J., Pacheco I.L., Pérez R., Escamilla A., Martínez-Moreno F.J., Martínez-Moreno A., Zafra R. (2018). Study of peritoneal macrophage immunophenotype in sheep experimentally infected with *Fasciola hepatica*. Vet. Parasitol..

[129] Escamilla A., Bautista M.J., Zafra R., Pacheco I.L., Ruiz M.T., Martínez-Cruz S., Méndez A., Martínez-Moreno A., Molina-Hernández V., Pérez J. (2016). *Fasciola hepatica* induces eosinophil apoptosis in the migratory and biliary stages of infection in sheep. Vet. Parasitol..

[130] Pacheco I.L., Abril N., Morales-Prieto N., Bautista M.J., Zafra R., Escamilla A., Ruiz M.T., Martínez-Moreno A., Pérez J. (2017). Th1/Th2 balance in the liver and hepatic lymph nodes of vaccinated and unvaccinated sheep during acute stages of infection with *Fasciola hepatica*. Vet. Parasitol..

[131] Hillyer G.V., Haroun E.T., Hernandez A., de Galanes M.S. (1987). Acquired resistance to *Fasciola hepatica* in cattle using a purified adult worm antigen. Am. J. Trop. Med. Hyg..

[132] Morrison C.A., Colin T., Sexton J.L., Bowen F., Wicker J., Friedel T., Spithill T.W. (1996). Protection of cattle against *Fasciola hepatica* infection by vaccination with glutathione S-transferase. Vaccine.

[133] Dalton J.P., McGonigle S., Rolph T.P., Andrews S.J. (1996). Induction of protective immunity in cattle against infection with *Fasciola hepatica* by vaccination with cathepsin L proteinases and with hemoglobin. Infect. Immun..

[134] Sexton J.L., Milner A.R., Panaccio M., Waddington J., Wijffels G., Chandler D., Thompson C., Wilson L., Spithill T.W., Mitchell G.F. (1990). Glutathione S-transferase. Novel vaccine against *Fasciola hepatica* infection in sheep. J. Immunol..

[135] Wijffels G.L., Salvatore L., Dosen M., Waddington J., Wilson L., Thompson C., Campbell N., Sexton J., Wicker J., Bowen F. (1994). Vaccination of sheep with purified cysteine proteinases of *Fasciola hepatica* decreases worm fecundity. Exp. Parasitol..

[136] Ramajo V., Oleaga A., Casanueva P., Hillyer G.V., Muro A. (2001). Vaccination of sheep against *Fasciola hepatica* with homologous fatty acid binding proteins. Vet. Parasitol..

[137] Martínez-Fernández A.R., Nogal-Ruiz J.J., López-Abán J., Ramajo V., Oleaga A., Manga-González Y., Hillyer G.V., Muro A. (2004). Vaccination of mice and sheep with Fh12 FABP from *Fasciola hepatica* using the new adjuvant/immunomodulator system ADAD. Vet. Parasitol..

[138] López-Abán J., Nogal-Ruiz J.J., Vicente B., Morrondo P., Diez-Baños P., Hillyer G.V., Martínez-Fernández A.R., Feliciano A.S., Muro A. (2008). The addition of a new immunomodulator with the adjuvant adaptation ADAD system using fatty acid binding proteins increases the protection against *Fasciola hepatica*. Vet. Parasitol..

[139] Buffoni L., Zafra R., Pérez-Ecija A., Martínez-Moreno F.J., Martínez-Galisteo E., Moreno T., Pérez J., Martínez-Moreno A. (2010). Immune response of goats immunised with glutathione S-transferase and experimentally challenged with *Fasciola hepatica*. Parasitol. Int..

[140] Wedrychowicz H., Kesik M., Kaliniak M., Kozak-Cieszczyk M., Jedlina-Panasiuk L., Jaros S., Plucienniczak A. (2007). Vaccine potential of inclusion bodies containing cysteine proteinase of *Fasciola hepatica* in calves and lambs experimentally challenged with metacercariae of the fluke. Vet. Parasitol..

[141] Dewilde S., Ioanitescu A.I., Kiger L., Gilany K., Marden M.C., Van Doorslaer S., Vercruysse J., Pesce A., Nardini M., Bolognesi M. (2008). The hemoglobins of the trematodes *Fasciola hepatica* and Paramphistomum epiclitum: A molecular biological, physico-chemical, kinetic, and vaccination study. Protein Sci. Publ. Protein Soc..

[142] Golden O., Flynn R.J., Read C., Sekiya M., Donnelly S.M., Stack C., Dalton J.P., Mulcahy G. (2010). Protection of cattle against a natural infection of *Fasciola hepatica* by vaccination with recombinant cathepsin L1 (rFhCL1). Vaccine.

[143] Maggioli G., Bottini G., Basika T., Alonzo P., Salinas G., Carmona C. (2016). Immunization with *Fasciola hepatica* thioredoxin glutathione reductase failed to confer protection against fasciolosis in cattle. Vet. Parasitol..

[144] Almeida M.S., Torloni H., Lee-Ho P., Vilar M.M., Thaumaturgo N., Simpson A.J.G., Tendler M. (2003). Vaccination against *Fasciola hepatica* infection using a *Schistosoma mansoni* defined recombinant antigen, Sm14. Parasite Immunol..

[145] Pérez-Caballero R., Buffoni L., Martínez-Moreno F.J., Zafra R., Molina-Hernández V., Pérez J., Martínez-Moreno Á. (2018). Expression of free radicals by peritoneal cells of sheep during the early stages of *Fasciola hepatica* infection. Parasit. Vectors.

[146] López-Abán J., Casanueva P., Nogal J., Arias M., Morrondo P., Diez-Baños P., Hillyer G.V., Martínez-Fernández A.R., Muro A. (2007). Progress in the development of *Fasciola hepatica* vaccine using recombinant fatty acid binding protein with the adjuvant adaptation system ADAD. Vet. Parasitol..

[147] Maggioli G., Acosta D., Silveira F., Rossi S., Giacaman S., Basika T., Gayo V., Rosadilla D., Roche L., Tort J. (2011). The recombinant gut-associated M17 leucine aminopeptidase in combination with different adjuvants confers a high level of protection against *Fasciola hepatica* infection in sheep. Vaccine.

[148] Buffoni L., Garza-Cuartero L., Pérez-Caballero R., Zafra R., Javier Martínez-Moreno F., Molina-Hernández V., Pérez J., Martínez-Moreno Á., Mulcahy G. (2020). Identification of protective peptides of *Fasciola hepatica*-derived cathepsin L1 (FhCL1) in vaccinated sheep by a linear B-cell epitope mapping approach. Parasit. Vectors.

[149] Buffoni L., Martínez-Moreno F.J., Zafra R., Mendes R.E., Pérez-Écija A., Sekiya M., Mulcahy G., Pérez J., Martínez-Moreno A. (2012). Humoral immune response in goats immunised with cathepsin L1, peroxiredoxin and Sm14 antigen and experimentally challenged with *Fasciola hepatica*. Vet. Parasitol..

[150] Mendes R.E., Pérez-Ecija R.A., Zafra R., Buffoni L., Martínez-Moreno A., Dalton J.P., Mulcahy G., Pérez J. (2010). Evaluation of hepatic changes and local and systemic immune responses in goats immunized with recombinant Peroxiredoxin (Prx) and challenged with *Fasciola hepatica*. Vaccine.

[151] Mendes R.E., Zafra R., Pérez-Ecija R.A., Buffoni L., Martínez-Moreno A., Tendler M., Pérez J. (2010). Evaluation of local immune response to *Fasciola hepatica* experimental infection in the liver and hepatic lymph nodes of goats immunized with Sm14 vaccine antigen. Mem. Inst. Oswaldo Cruz.

[152] Zafra R., Buffoni L., Martínez-Moreno A., Pérez-Ecija A., Martinez-Moreno F.J., Pérez J. (2008). A study of the liver of goats immunized with a synthetic peptide of the Sm14 antigen and challenged with *Fasciola hepatica*. J. Comp. Pathol..

[153] Zafra R., Pérez-Écija R.A., Buffoni L., Moreno P., Bautista M.J., Martínez-Moreno A., Mulcahy G., Dalton J.P., Pérez J. (2013). Early and late peritoneal and hepatic changes in goats immunized with recombinant cathepsin L1 and infected with *Fasciola hepatica*. J. Comp. Pathol..

[154] Pérez-Ecija R.A., Mendes R.E., Zafra R., Buffonni L., Martínez-Moreno A., Pérez J. (2010). Pathological and parasitological protection in goats immunised with recombinant cathepsin L1 and challenged with *Fasciola hepatica*. Vet. J. Lond. Engl. 1997.

[155] Ortega-Vargas S., Espitia C., Sahagún-Ruiz A., Parada C., Balderas-Loaeza A., Villa-Mancera A., Quiroz-Romero H. (2019). Moderate protection is induced by a chimeric protein composed of leucine aminopeptidase and cathepsin L1 against *Fasciola hepatica* challenge in sheep. Vaccine.

[156] Villa-Mancera A., Reynoso-Palomar A., Utrera-Quintana F., Carreón-Luna L. (2014). Cathepsin L1 mimotopes with adjuvant Quil A induces a Th1/Th2 immune response and confers significant protection against *Fasciola hepatica* infection in goats. Parasitol. Res..

[157] Villa-Mancera A., Alcalá-Canto Y., Olivares-Pérez J., Molina-Mendoza P., Hernández-Guzmán K., Utrera-Quintana F., Carreón-Luna L., Olmedo-Juárez A., Reynoso-Palomar A. (2021). Vaccination with cathepsin L mimotopes of *Fasciola hepatica* in goats reduces worm burden, morphometric measurements, and reproductive structures. Microb. Pathog..

[158] Villa-Mancera A., Alcalá-Canto Y., Reynoso-Palomar A., Olmedo-Juárez A., Olivares-Pérez J. (2021). Vaccination with cathepsin L phage-exposed mimotopes, single or in combination, reduce size, fluke burden, egg production and viability in sheep experimentally infected with *Fasciola hepatica*. Parasitol. Int..

[159] Wesołowska A., Zawistowska-Deniziak A., Norbury L.J., Wilkowski P., Januszkiewicz K., Pyziel A.M., Zygner W., Wędrychowicz H. (2016). Immune responses in rats and sheep induced by a DNA vaccine containing the phosphoglycerate kinase gene of *Fasciola hepatica* and liver fluke infection. Acta Parasitol..

[160] Wesołowska A., Basałaj K., Zawistowska-Deniziak A., Januszkiewicz K., Kozak Ljunggren M., Jedlina L., Wędrychowicz H. (2019). The failure of a DNA prime/protein boost regime and CTLA-4 mediated targeting to improve the potency of a DNA vaccine encoding *Fasciola hepatica* phosphoglycerate kinase in sheep. Vet. Immunol. Immunopathol..

[161] Norbury L.J., Basałaj K., Zawistowska-Deniziak A., Sielicka A., Wilkowski P., Wesołowska A., Smooker P.M., Wędrychowicz H. (2018). Intranasal delivery of a formulation containing stage-specific recombinant proteins of *Fasciola hepatica* cathepsin L5 and cathepsin B2 triggers an anti-fecundity effect and an adjuvant-mediated reduction in fluke burden in sheep. Vet. Parasitol..

